# Quantum Dots-siRNA Nanoplexes for Gene Silencing in Central Nervous System Tumor Cells

**DOI:** 10.3389/fphar.2017.00182

**Published:** 2017-04-04

**Authors:** Guimiao Lin, Ting Chen, Jinyun Zou, Yucheng Wang, Xiaomei Wang, Jiefeng Li, Qijun Huang, Zicai Fu, Yingying Zhao, Marie Chia-Mi Lin, Gaixia Xu, Ken-Tye Yong

**Affiliations:** ^1^Department of Physiology, School of Basic Medical Sciences, Shenzhen University Health Sciences CenterShenzhen, China; ^2^Key Laboratory of Optoelectronics Devices and Systems of Ministry of Education/Guangdong Province, College of Optoelectronic Engineering, Shenzhen UniversityShenzhen, China; ^3^School of Electrical and Electronic Engineering, Nanyang Technological UniversitySingapore, Singapore

**Keywords:** quantum dots, RNA interfering, gene delivery, human telomerase reverse transcriptase, glioblastoma

## Abstract

RNA interfering (RNAi) using short interfering RNA (siRNA) is becoming a promising approach for cancer gene therapy. However, owing to the lack of safe and efficient carriers, the application of RNAi for clinical use is still very limited. In this study, we have developed cadmium sulphoselenide/Zinc sulfide quantum dots (CdSSe/ZnS QDs)-based nanocarriers for *in vitro* gene delivery. These CdSSe/ZnS QDs are functionalized with polyethyleneimine (PEI) to form stable nanoplex (QD-PEI) and subsequently they are used for siRNA loading which specially targets human telomerase reverse transcriptase (TERT). High gene transfection efficiency (>80%) was achieved on two glioblastoma cell lines, U87 and U251. The gene expression level (49.99 ± 10.23% for U87, 43.28 ± 9.66% for U251) and protein expression level (51.58 ± 7.88% for U87, 50.69 ± 7.59% for U251) of TERT is observed to decrease substantially after transfecting the tumor cells for 48 h. More importantly, the silencing of TERT gene expression significantly suppressed the proliferation of glioblastoma cells. No obvious cytotoxicity from these QD-PEI nanoplexes were observed over at 10 times of the transfected doses. Based on these results, we envision that QDs engineered here can be used as a safe and efficient gene nanocarrier for siRNA delivery and a promising tool for future cancer gene therapy applications.

## Introduction

Glioblastoma is the most common and aggressive primary central nervous system (CNS) tumor. The prognosis of glioblastoma remains very poor to date and highly lethal with the average survival time of patients with glioblastoma is estimated to be 1 year (Redmond and Mehta, 2015). Although, standard therapeutic approaches have been used to combat this disease, current therapies are still insufficient in treating the glioblastoma patients (Adamson et al., 2009). Glioblastoma is highly infiltrative and relatively resistant to conventional therapeutic approaches. Therefore, the development of new, novel and effective therapeutic strategy is of utmost importance to cure this deadly disease.

RNA interference (RNAi) is a promising technique with great opportunity to battle with cancer (Mansoori et al., 2014). The discovery of RNAi has generated profound therapeutic promises for gene therapy of cancer using small interfering RNA (siRNA) (Seyhan, 2011). The process of RNAi is complex and initiated by double-stranded siRNA with a typical length of around 21 base pairs. The siRNA is sequenced to the target gene and able to arouse a sequence-specific gene silencing in post-transcription level. In the past years, RNAi-based cancer gene therapy has been extensively explored (Maduri, 2015; Gan et al., 2016). The success of RNAi therapeutic approach depends on two important factors, namely, efficient therapeutic targets and the delivery efficiency.

Telomerase is an attractive target for cancer gene therapy due to its high prevalence in almost all of the tumor cells (Tian et al., 2010). The important function of telomerase is to prolong the life span of cells by maintaining the telomere length (Lu et al., 2013). Activation of telomerase is the critical step in oncogenesis and cellular immortality. Human telomerase reverse transcriptase (TERT) is the catalytic protein subunit of telomerase and it is very critical for the activity of telomerase in tumor cells (Osterhage and Friedman, 2009). The dysfunction of TERT will prevent the long-term survival of tumor cells and promote cell apoptosis and death. Based on these known facts, considerable new strategies including RNAi have been explored to target the TERT gene for cancer gene therapy (Xie et al., 2011).

In addition to ideal therapeutic target, safe and efficient delivery carrier is another crucial factor for successful gene therapy. Naked siRNAs is easy to be degraded by RNase, leading to its short half-life. More importantly, because of it negatively charged nature, free siRNA is unable to penetrate the cell membrane (Whitehead et al., 2009). To address this issue, a variety of carriers have been extensively utilized to facilitate the delivery of siRNA into cytoplasm (Shi et al., 2014; Dim et al., 2015; Li et al., 2015; Fitzgerald et al., 2016; Warashina et al., 2016). Among them, fluorescent quantum dots (QDs) have been investigated as potential carriers for the loading of gene molecules owing to their stable chemical properties and large surface area (Jung et al., 2010; Zhao et al., 2013; Wang et al., 2015). What's more important, QDs have unique optical features such as high resistance to photobleaching, size-tunable fluorescent peaks and a broad excitation profile with narrow emission spectra (Bruchez et al., 1998). These exceptional optical properties make them useful for real-time monitoring of the siRNA delivery process *in situ*.

In this study, we developed cadmium sulphoselenide/Zinc sulfide quantum dots (CdSSe/ZnS QDs)-based nanocarriers for *in vitro* gene delivery. These CdSSe/ZnS QDs are conjugated with Polyethyleneimine (PEI) to form stable nanoplex (QD-PEI) with enhancing zeta potential and subsequently used for siRNA loading through electrostatic interaction. For genetic therapy of glioblastoma, we designed siRNA sequences specifically targeting TERT gene. In our experiments, two different glioblastoma cell lines (U87 and U251) were used for comparison studies. We found that the delivery of siRNA by QD-PEI nanoplex was effective and the gene expression of TERT was successfully suppressed. We also observed the down-regulation of TERT gene had inhibited the proliferation of the glioblastoma cells and we envisioned that such engineered approach might serve as a new and effective way for combating glioblastoma gene therapy.

## Materials and methods

### Preparation and characterization of QDs

CdSSe/ZnS QDs were purchased from Najingtech Company and it is terminated with COOH surface groups by coating with a polymer layer. The fluorescence spectra of CdSSe/ZnS QDs were determined by a spectrophotometer (F-4600, Hitachi, Japan). The hydrodynamic size distribution of the QDs was obtained using a dynamic light scattering (DLS) machine (Zetasizer Nano ZS, Malvern, UK). The morphology images of CdSSe/ZnS QDs were obtained with a transmission electron microscope (TEM) (Tecnai G2 F20 S-TWIN, FEI, USA) operating at an accelerating voltage of 200 kV at room temperature.

### Cell culture

The human neuroglioma cells U87 and U251 were obtained from American Type Culture Collection (ATCC) and maintained in Dulbecco's Modified Eagle's Medium (DMEM) (Gibco) and RMPI 1640 medium respectively. The culture mediums were supplemented with 10% fetal bovine serum (FBS) (Gibco), penicillin (100U, Gibco) and streptomycin (100U, Gibco). Cells were kept at 37°C in a humidified incubator with 5% CO_2_.

### Preparation of QD-PEI particles

Branched PEI reagent with 25 KDa molecular weight (Sigma, US) was used to modify the surface of the QDs. Briefly, 50 μL of QDs solution (1 mg/mL) was mixed with 250 μL of PEI solution at different concentrations (0.05, 0.1, 0.25, and 0.5 mg/mL, in deionized water), followed by short sonication and vortexing for 20 min. Then the QD-PEI particles were collected by centrifugation at 14,000 rpm for 10 min. The resulting QD-PEI particles were dispersed in 200 μL of DEPC-treated water while the aggregates were removed by spinning down at 2,000 rpm for 1 min.

### Transfection

Scramble siRNA, TERT siRNA, and TERT siRNA^FAM^ were purchased from Genepharma Company, China. The siRNAs with FAM probe were used for laser scanning confocal imaging analysis and flow cytometry assay. The day before transfection, cells were planted onto 6-well plates in medium without antibiotics to give 30–50% density. For siRNA loading, QD-PEI particle (100 μg/mL, 40 μL) was mixed with siRNAs (10 μM, 10 μL) with gentle vortexing, and the mixture was left undisturbed for 30 min. Before transfection, the culture medium was replaced by fresh medium without FBS, and the above mentioned QD-PEI-siRNA mixture was then added to the medium with a final volume of 2 mL (2 μg/mL QD-PEI). Scramble siRNA with non-targeted siRNA sequences was used as a negative control at the same dosage. In a parallel experiment, Lipofectamine2000 (Invitrogen), a frequently reported commercial transfection reagent was used as positive control. The transfection efficiency was observed at 4 h post-transfection, and the gene and protein expression was determined at 48 h post-transfection.

### Laser scanning confocal imaging

Four hours after transfection, the *in vitro* confocal images were obtained using a laser scanning confocal microscope (LSCM) (TCS SP5, Leica, DEU). The FAM signals from siRNAs were utilized to monitor the siRNAs. Before imaging, the cell medium was removed and cells were washed with phosphate-buffered saline (PBS) twice, fixed with formaldehyde (4%) for 10 min. Then the formaldehyde solution was removed and the nuclei were stained with DAPI (Sigma) for 5 min. To image the cells, filter sets for DAPI (excitation: 405 nm; emission: 450/50 nm) and FAM (excitation: 488 nm; emission: 525/50 nm) were applied, respectively.

### Flow cytometry

For the flow cytometry assays, cells were washed twice with PBS solution and harvested by trypsin (Gibco). The FAM signals from siRNAs were utilized to determine the transfection efficiency quantitatively. After trypsinization, the cells were resuspended in 500 μL PBS solutions and analyzed immediately by a flow cytometry machine (FACSAria II, BD, USA).

### Quantitative gene expression analysis

Forty-eight hours after transfection, total RNA of U87 and U251 cells was extracted using TRIzol reagent (Invitrogen) and quantitated by a spectrophotometer (Epoch, BioTek, USA). Total RNA (1 μg) was reverse transcribed to cDNA using one-step reverse transcriptase kit (TransGen) according to the manufacturer's instructions. Real-time polymerase chain reaction (TransGen) was then performed by a PCR machine (CFX96, Bio-Rad, USA). The TERT gene expression level was normalized to the expression of a commonly used housekeeping gene, Glyceraldehyde-3-phosphate dehydrogenase (GAPDH). Primers used were as follows:

TERT: 5′-AGAGTGCCTTGACGATACAGC-3′ (sense), 5′-ACAAAGAAAGCCCTCCCCAGT-3′ (antisense); GAPDH: 5′-ACCACAGTCCATGCCATCAC-3′ (sense), GAPDH 5′-TCCACCACCCTGTTGCTGTA-3′ (antisense).

### Western blot analysis

Forty-eight hours after transfection, total proteins of U87 and U251 cells were extracted using M-PER Mammalian Protein Extraction Reagent (Thermo) and quantitated by BCA Reagent (Tiangen). Proteins (16 μg) were electrophoresed through 8% (w/v) polyacrylamide, 0.5% (w/v) SDS gels. Proteins were transferred to nitrocellulose membrane (Millipore), followed by blocking with 5% (w/v) non-fat milk in TBST buffer for 1 h at room temperature, incubatingwith TERT and β-tubulin antibody (1:1,000; GeneTex) overnight at 4°C. Primary antibody was detected using horseradish peroxidase-conjugated goat anti-rabbit IgG-HRP (1:1,000; NeoBioscience). Blots were developed with clarity western ECL substrate kit (BioRad, USA) and imaged by chemiluminescence imaging system (Qinxiang science instruments, China). The relative protein expression level of TERT was normalized to the expression of β-tubulin. The relative expression level was expressed as a percentage, assigning the relative expression level of TERT in Blank group as 100%.

### Cell viability

Cell viability was measured by the MTS (Promega) assay. Cells were seeded in 96-well plates (5 × 10^3^ cells/well) and incubated with different concentrations of QD-PEI solutions for 24 or 48 h. MTS solution in PBS buffer was added into the cells and the cells were incubated for 4 h at 37°C with 5% CO_2_. The cells were gently shaked for 5 min and absorbance was measured with a microplate reader (Epoch, Bio-Tek, USA) at a wavelength of 490 nm. The cell viability was calculated by normalizing the absorbance of the QDs-treated well against that of the control well. The cell viability was expressed as a percentage, assigning the viability of control cells as 100%.

### Statistical analysis

All data were presented as mean ± SD. The results were analyzed via one-way ANOVA with a *post-hoc* Dunnett test to determine statistical significance. All statistical calculations were performed with the SPSS 11.0 software package. A *p* value less than 0.05 was regarded as statistically significant difference.

## Results

**Figure 1:**
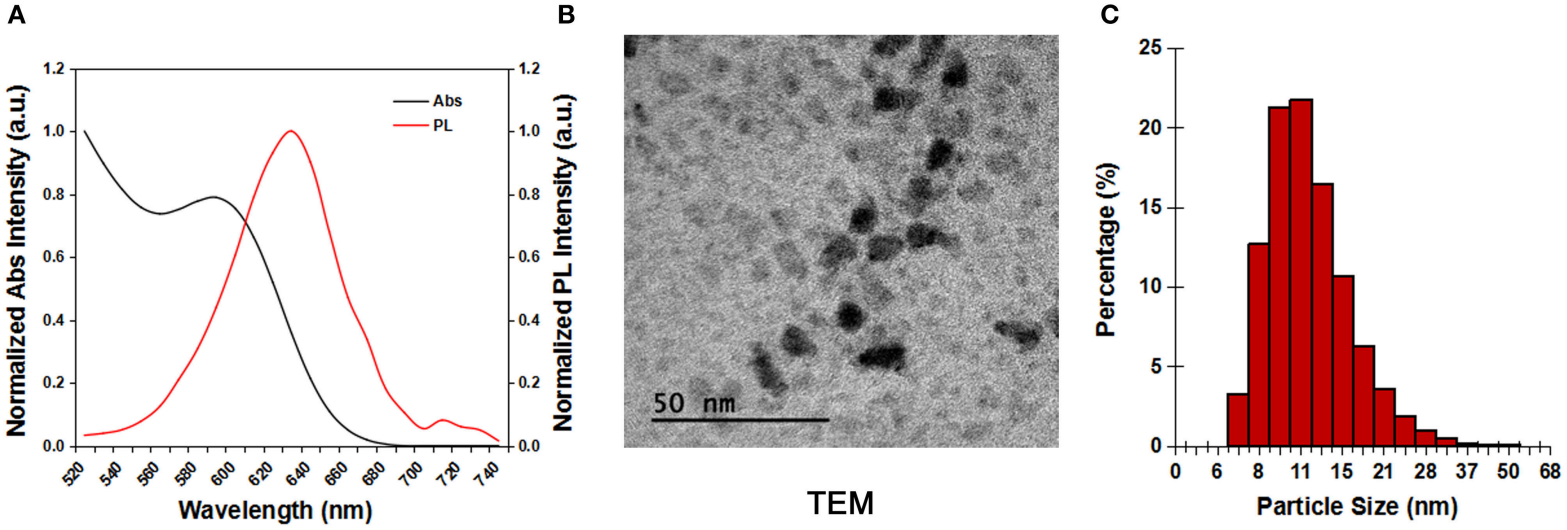
**The characterization of the CdSSe/ZnS QDs. (A)** Absorption and photoluminescent spectra of the CdSSe/ZnS QDs. **(B)** TEM image of the CdSSe/ZnS QDs. **(C)** DLS measurement of the CdSSe/ZnS QDs in deionized water.

Figure 1A shows the absorption and photoluminescence spectra of the dispersion of carboxyl-terminated CdSSe/ZnS QDs in water. The QDs exhibit an absorption peak at 590 nm and an emission peak at 630 nm. Due to the presence of carboxylic groups on the shells, the CdSSe/ZnS QDs were negatively charged with zeta potential value of −45.6 mV. The TEM image of the CdSSe/ZnS QDs showed that the nanocrystals are highly monodispersed (Figure 1B) in their size distribution. Figure 1C shows the hydrodynamic size distribution of the QDs characterized by DLS. The QDs have a narrow size distribution with a hydrodynamic diameter of 12.97 ± 4.62 nm.

**Figure 2:**
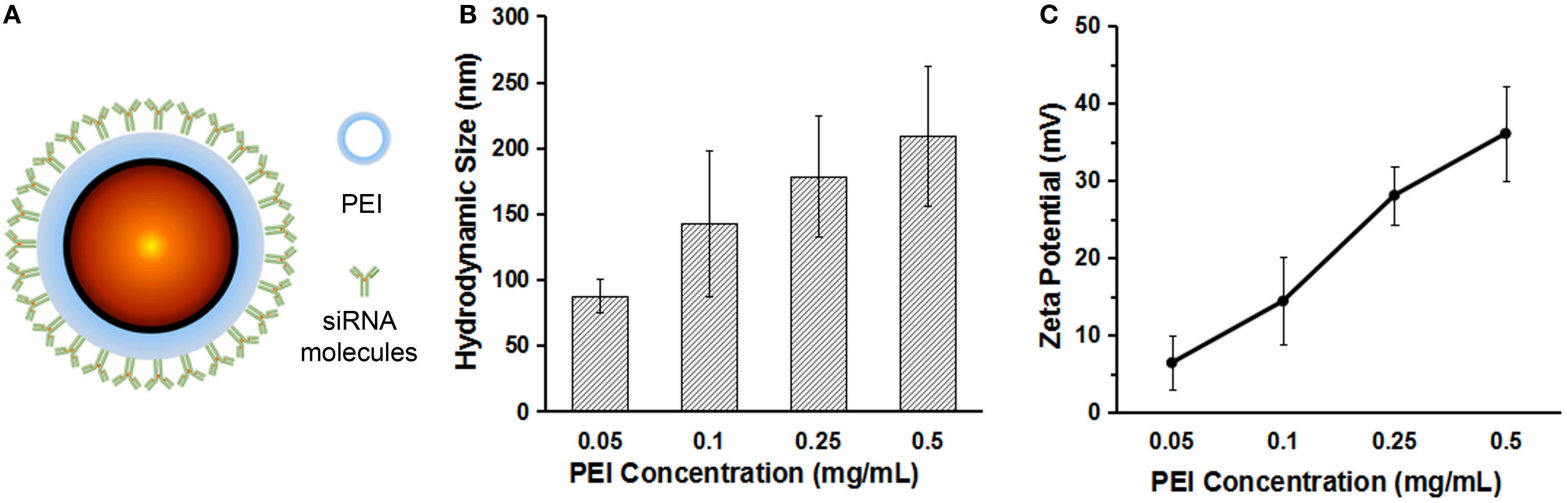
**The characterization of the QD–PEI nanoparticle. (A)** Scheme of the QD–PEI-siRNA nanoplexes. **(B)** Hydrodynamic size and **(C)** surface zeta potential of the QD-PEI clusters prepared using different PEI concentrations. Values are means ± SD, *n* = 3.

For siRNA loading, PEI was used to modify the surface of the QDs. Figure 2A shows the scheme of the QD–PEI-siRNA nanoplexes. By changing the PEI concentration, the QD-PEI clusters can be prepared with different hydrodynamic sizes (87.8~209.4 nm), and the surface zeta potential can also be tailored ranging from +6.44 to +36.10 mV (Figures 2B,C). The high zeta potential is crucial for siRNA loading since the siRNAs are negatively charged by nature. The negatively charged siRNAs can be conjugated to the QD-PEI nanoparticles through electrostatic interaction. Finally, PEI solution of 0.5 mg/mL was used to modify the QDs, and QD-PEI solution at final concentration of 2 μg/mL was utilized for siRNAs transfection.

**Figure 3:**
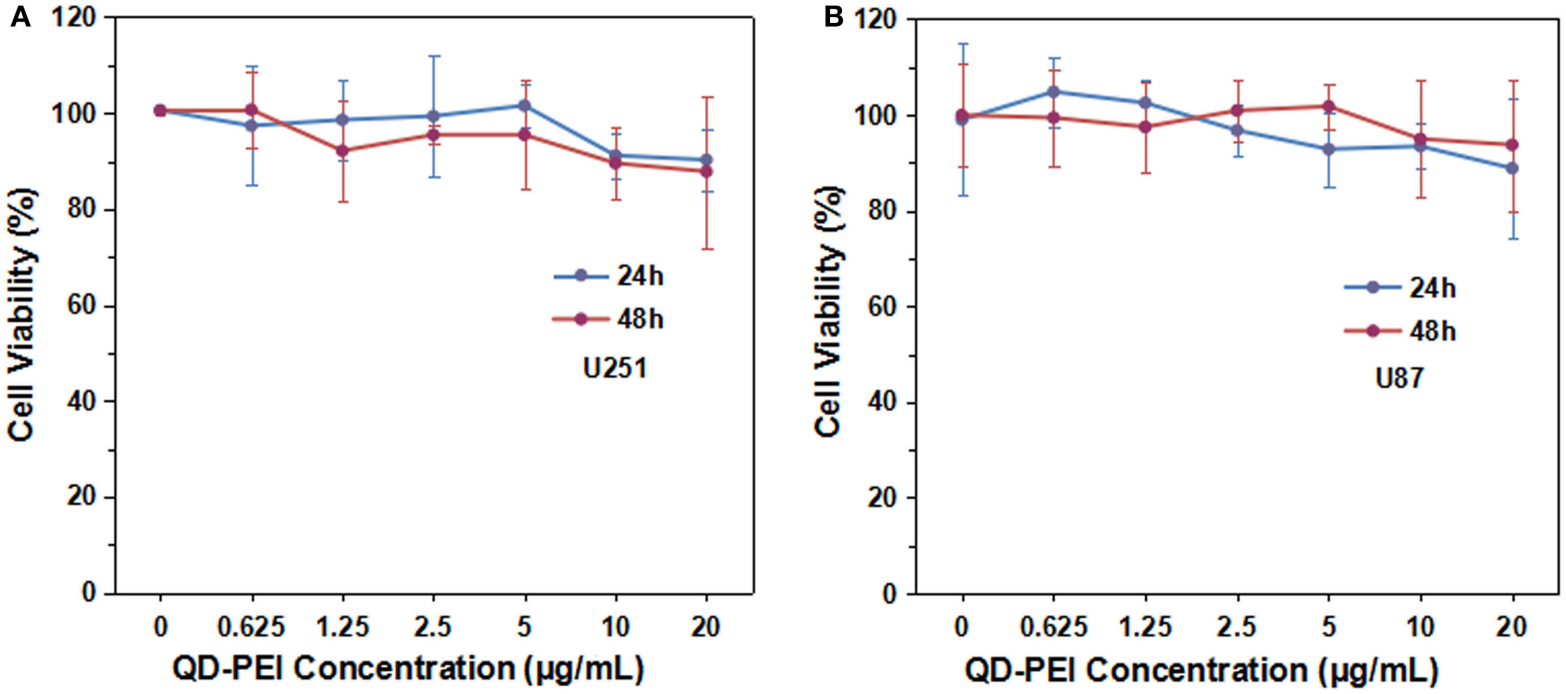
**Cytotoxicity tests of QD-PEI formulation by MTS assays. (A)** U251 cells and **(B)** U87 cells were treated with different concentrations of QD-PEI formulation for 24 or 48 h. Values are means ± SD, *n* = 5.

To construct an efficient gene nanocarrier, the prepared nanoparticles should be biocompatible. In order to determine the safe dosage range of the QD-PEI formulation, *in vitro* cell viability studies were carried out by using MTS assay. The U251 and U87 cells were treated with various doses of QD-PEI formulation for 24 or 48 h. Figure 3 shows that over 90% cell viability was maintained when the applied doses of QD-PEI formulation ranged from 0.625 to 20 μg/mL (within 24 and 48 h). According to our results, the QD-PEI formulation should have limited cytotoxicity effects on the cells if the concentration of the nanoplex is below 20 μg/mL (10 times of transfection concentration).

**Figure 4:**
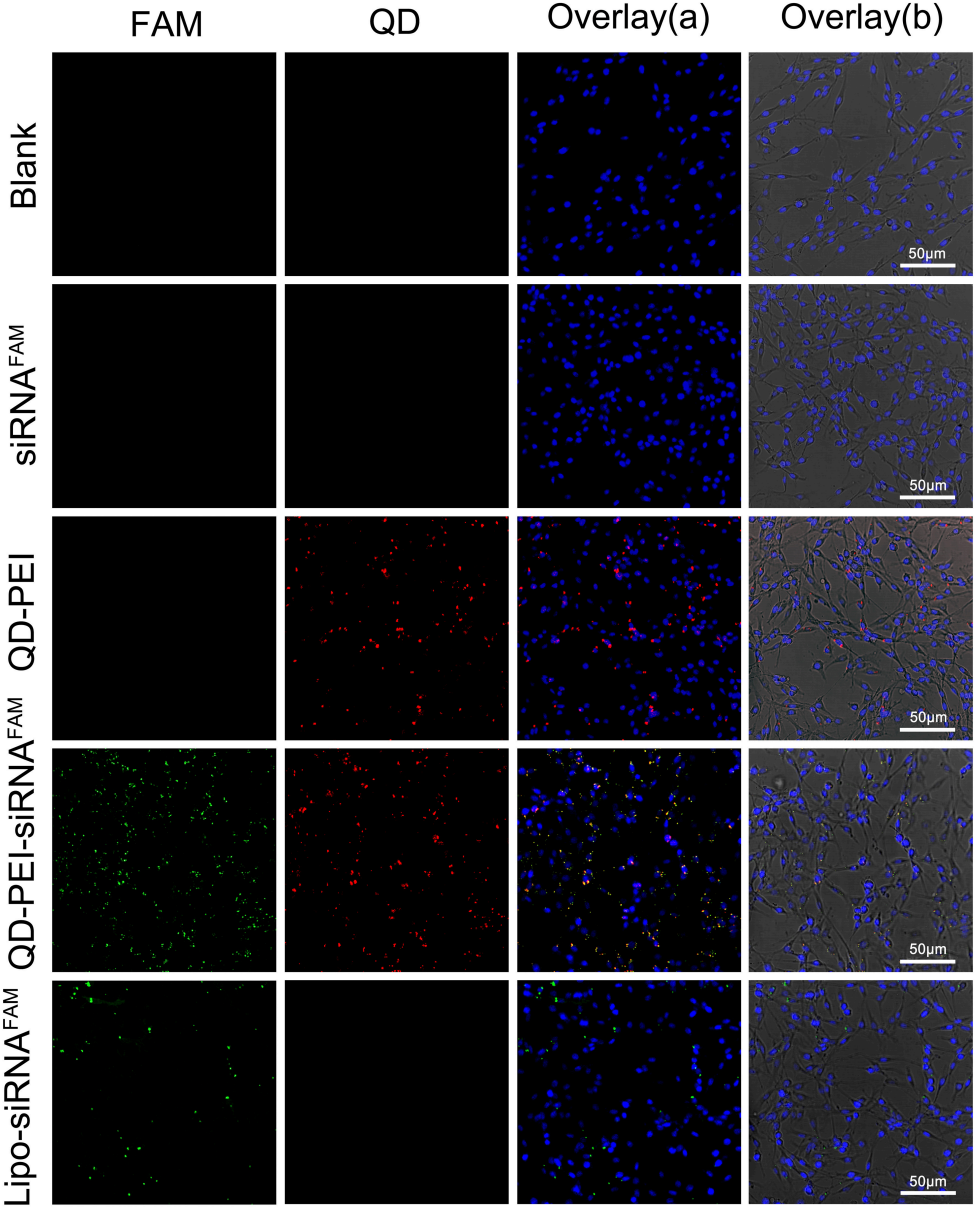
**Laser confocal images of U251 cells treated with different nanoformulations**. (I) Blank, (II) free siRNA^FAM^, (III) QD-PEI, (IV) QD-PEI-siRNA^FAM^, and (V) Lipo-siRNA^FAM^ for 4 h. Cell nucleus is stained with DAPI (in blue), signals from QD are assigned in red and signals from FAM are assigned in green.

The QD-PEI particles were then served as nanocarriers to deliver siRNAs which target human TERT gene in human neuroglioma cell lines, U87 and U251. It is worth mentioning that TERT is the catalytic protein subunit of telomerase, and it is important for maintaining the activity of telomerase in cancer cells. Figure 4 shows the confocal images of tumor cells of 4 h post-transfection with different nanoplexes. The fluorescent signal from TERT siRNAs was detectable in cells treated with QD-PEI-siRNA formulation, where the TERT siRNAs were labeled with fluorescence FAM. Similar results were also observed in the positive control group where the commercially available transfection reagent Lipofectamin2000 was used to deliver the siRNAs. The results demonstrate that the TERT siRNAs were successfully delivered into the cells by functionalizing them to QD-PEI formulation. In contrast, no fluorescence signal is detected in the cells treated with naked siRNAs, suggesting that the free siRNAs are unable to penetrate the cell membrane in the absence of delivery nanovectors.

**Figure 5:**
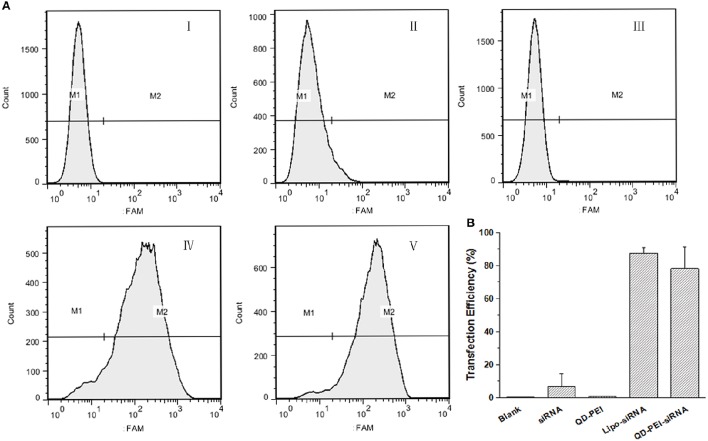
**Transfection efficiency of U251 cells determined by flow cytometry analysis. (A)** Representative pictures, where cells were treated with (I) Blank, (II) free siRNA, (III) QD-PEI, (IV) Lipo-siRNA, and (V) QD-PEI-siRNA. **(B)** Percentage of cells transfected after 4-h treatment, evaluated from experiments shown in **(A)**. Values are means ± SD, *n* = 4.

In order to further quantitatively evaluate the transfection efficiency of siRNAs by QD-PEI, flow cytometry assay was performed. Figure 5 shows the representative images of the FAM fluorescence intensity in U251 cells treated with different nanoplexes for 4 h. Consistent with the confocal imaging analysis, the result shows that almost no fluorescence signal is detected for the cells treated with naked TERT siRNA^FAM^ (6.78 ± 7.85%) and QD-PEI alone (0.27 ± 0.24%). As a comparison, cells treated with Lipo-siRNA^FAM^ and QD-PEI-siRNA^FAM^ showed strong fluorescence signals, suggesting the abundant accumulation of siRNA^FAM^ in the tumor cells. The fraction of the tumor cells with positive FAM fluorescence signals for the cells treated with QD-PEI-siRNA^FAM^ is estimated to be 80%. These results demonstrated that the QD-PEI nanoplex formulation can be utilized as an efficient siRNA delivery nanovector.

**Figure 6:**
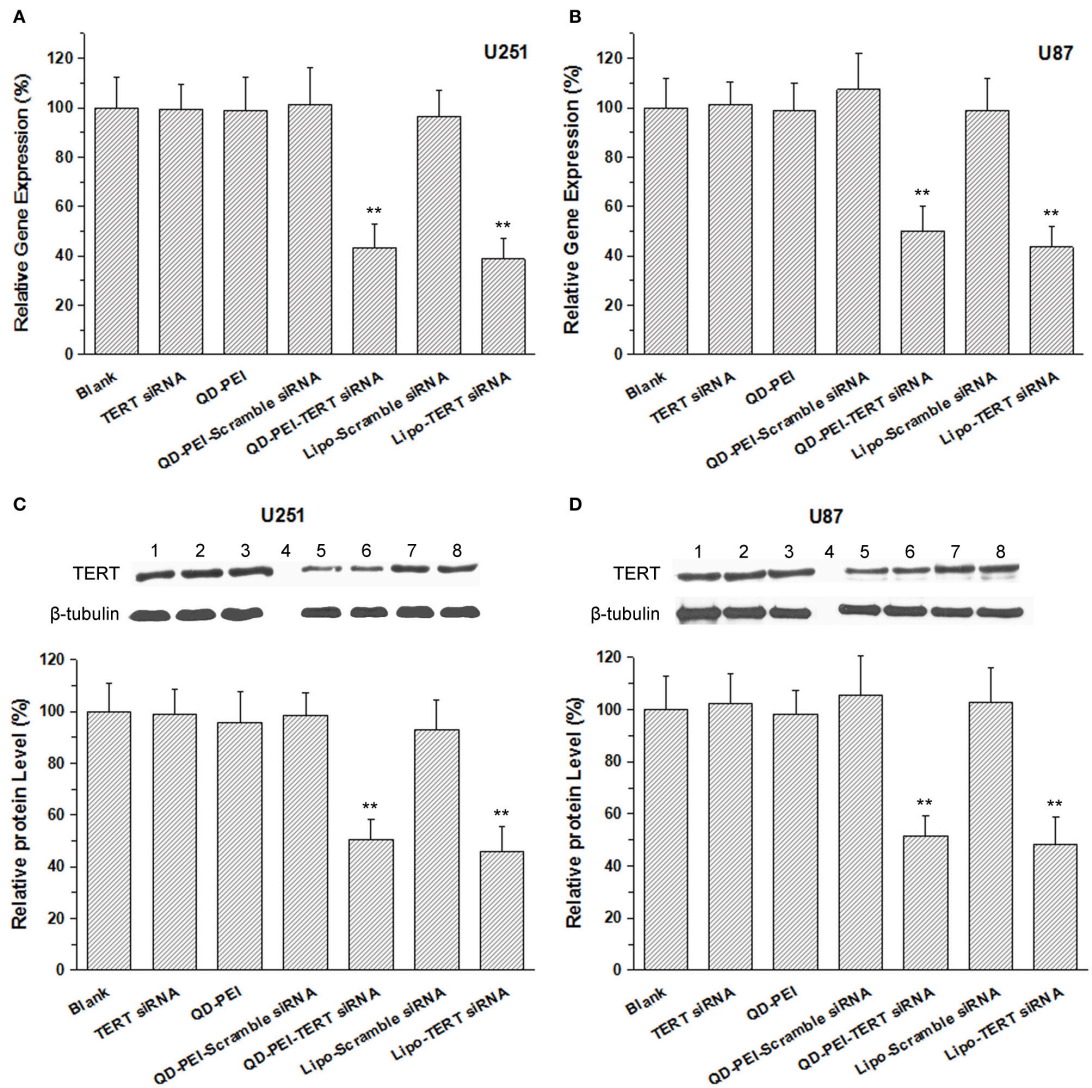
**The mRNA and protein expression levels of the TERT gene**. **(A,B)** TERT mRNA relative expression levels in **(A)** U251 cells and **(B)** U87 cells determined by real-time PCR. **(C,D)** Representative images of TERT protein expression and relative expression levels in **(C)** U251 cells and **(D)** U87 cells determined by Western Blot analysis. Lane1-8 refer to Blank, TERT siRNA, QD-PEI, Protein marker, QD-PEI-TERT-siRNA, Lipo-TERT-siRNA, QD-PEI-Scramble siRNA, Lipo- Scramble-siRNA. Values are means ± SD, *n* = 3. Values are means ± SD, *n* = 5. ^**^p < 0.01 vs. Blank.

To confirm that we had a successful knocking down process *in vitro*, the mRNA and protein expression level of the targeted TERT gene was determined by real-time PCR and Western blot analysis. Figures 6A,B shows the quantitative expression of TERT mRNA in U251 and U87 cells transfected with different formulations for 48 h. The results demonstrate that the cells treated with TERT siRNA, QD-PEI, QD-PEI-Scramble siRNA and Lipo-Scramble siRNA exhibited minimal suppressions upon comparing to the cells without any treatment. In contrast, the expression level of TERT mRNA in cells transfected with QD-PEI-TERT siRNA nanoplex was found to be significantly suppressed (*p* < 0.01). The mRNA expression level of QD-PEI-TERT siRNA was 43.28 ± 9.66% for U251 cells and 49.99 ± 10.23% for U87 cells. No difference was observed in the mRNA expression between cells treated with QD-PEI-TERT siRNA and cells treated with Lipo-TERT siRNA (*p* > 0.05). Consistent result for TERT protein expression was obtained by Western blot analysis (Figures 6C,D). The TERT protein expression level of QD-PEI-TERT siRNA was determined to be 50.69 ± 7.59% for U251 cells and 51.58 ± 7.88% for U87 cells. These results suggest that the formulation presented here is effective for gene silencing and can be served as a good platform for gene therapy applications.

**Figure 7:**
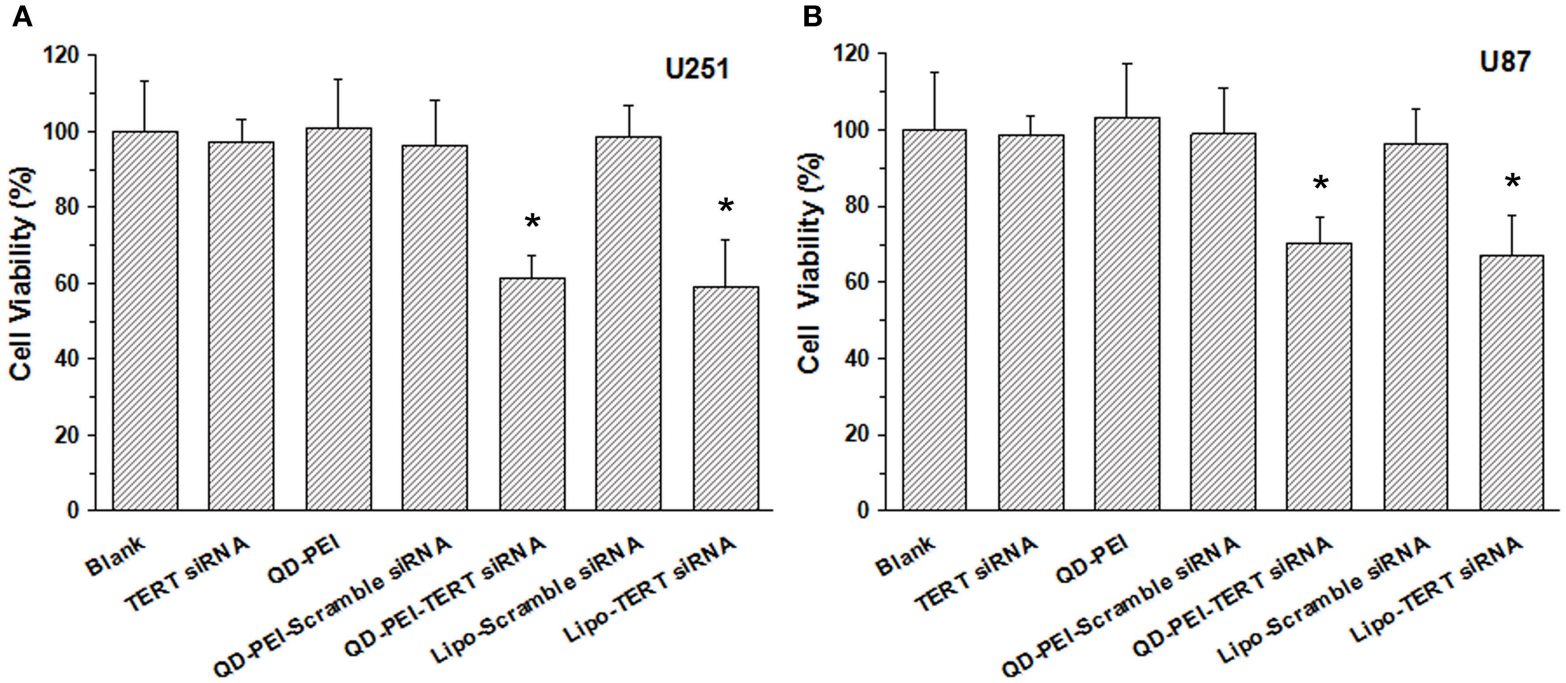
**Cell viabilites of cells treated with different nanoformulations**, **(A)** U251 cells and **(B)** U87 cells. Values are means ± SD, *n* = 5. ^*^p < 0.05 vs. Blank.

In addition to the gene silencing efficiency, to evaluate the targeted gene therapeutic effects of the TERT siRNAs delivered by QD-PEI formulation, the viability of the cells with 48-h treatment were examined by MTS assays. Both U251 and U87 cells were treated with different nanoformulations for 48 h and their corresponding cell viabilities were evaluated. As shown in Figure 7, no significant difference was observed in the cell proliferation ability between the Blank, TERT siRNA, QD-PEI, and QD-PEI-Scramble siRNA groups. On the contrary, an evident decrease in the cell viabilities was observed for the cells treated with QD-PEI-TERT siRNA formulation upon comparing to the cells without any treatment (*p* < 0.05). No difference was observed in the cell viability between cells treated with QD-PEI-TERT siRNA and cells treated with Lipo-TERT siRNA (*p* > 0.05). This indicates that TERT siRNAs delivered by QD-PEI particle is an efficient and promising strategy to suppress the proliferation of both the U251 and U87 human neuroglioma cells.

## Discussion

The aggressiveness of glioblastoma has made it the most deadly CNS tumor (Adamson et al., 2009). The siRNAs-based RNAi technology has brought potential promises for patients to fight against this deadly disease by gene therapy approach (Gan et al., 2016). Great efforts have been made by the medicine research community to seek effective gene targets for treating glioblastoma and several strategies have been proposed and identified (Zorzan et al., 2015). In this study, anti-tumor therapeutics targeting TERT was employed as a unique therapy strategy due to the high prevalence of telomerase in most of the tumor cells (Ruden and Puri, 2013).

Besides therapeutics target, an ideal delivery vehicle with high gene knocking down efficiency is still in urgent need for RNAi-based gene therapy since naked siRNAs are extremely unstable and negatively charged (Whitehead et al., 2009). Previously, viral vectors have been demonstrated as promising gene delivery carriers owing to their high transfection efficiency (Couto and High, 2010). However, along with the rise of concern on biosafety issues and immune response problems, the application of viral vectors was severely impeded (Chira et al., 2015). As a result, non-viral vehicles for siRNA delivery have emerged as an alternative method and it has been investigated by many research groups around the globe (De Laporte et al., 2006; Chira et al., 2015). Compared with viral vectors, non-viral vectors have several advantages such as ease of design and synthesis, flexible surface modifications, tremendous potential for optimization and enhanced biosafety (Chen and Huang, 2008; Wong et al., 2012; Lin et al., 2013). More importantly, these non-viral vectors are potential candidates to be translated to clinical usage (Merkel et al., 2014).

Previously, several studies have already shown the application prospects of QDs in gene transfer and therapy (Chen et al., 2005; Jung et al., 2010; Li et al., 2011, 2012; Zhao et al., 2013). QDs-based methods include the advantages of real-time imaging and monitoring the intracellular trafficking of small molecules within the cells. Chen et al. demonstrated a method of monitoring and improving siRNA delivery using water-dispersible and mercaptoacetic acid-coated (MAA) QDs where their surface was modified with polyethylene glycol (PEG) (Chen et al., 2005). Li et al. reported the delivery of QD-siRNA nanoplexes into SK-N-SH cell line for BACE1 gene silencing and effective monitoring the spatiotemporal distribution and dynamics of QDs by real-time intracellular imaging (Li et al., 2012). In this study, carboxyl–terminated CdSSe/ZnS QDs were designed for the delivery of siRNAs into the glioblastoma cells. Because of the carboxylic groups terminated on their surface, the CdSSe/ZnS QDs have a negatively charged surface and the zeta potential is determined to be −45.6 mV. To increase the value of zeta potential, the QDs were further functionalized with PEI which was needed for gene transfection application (Lu et al., 2015; Chen et al., 2016). Owing to their large density of positively charged amino groups, PEI has a strong ability for DNA binding and cell adhesion. After functionalizing the QDs with PEI, the zeta potential of QD-PEI nanoformulation was increased. This is desirable for gene delivery because it enhances the efficiency of siRNA loading through the electrostatic interaction. It is worth mentioning that higher charge density will generally provide higher loading efficiency of the oppositely charged siRNA molecules, but excess charge on the QDs surface will result in the decrease of the siRNAs release owing to the strong binding interaction between the QDs and siRNA molecules (Wang et al., 2015). Wang et al. suggested that a zeta potential value ranged from +20 to +40 mV is optimal for the formation of nanoplex and allowing them to be effectively delivering siRNAs to the cells (Wang et al., 2015). Consistently, in this study, the zeta potential of nanoplex of CdSSe/ZnS QDs is estimated to be +36 mV, which is suitable for the siRNA delivery.

After the transfection, the gene transfection efficiency, the gene expression level, as well as the cell viability were determined. We found that the delivery of siRNAs by QD-PEI nanocarrier was highly effective and the proliferation of tumor cells was successfully suppressed, suggesting that QD-PEI-siRNA complexes were able to escape from the endosomes and releasing the siRNAs into the cytoplasm. Also, efficient gene silencing and cell proliferation inhibition was achieved by TERT mRNA targeting, which thereby inducing the specific siRNA-induced gene silencing. To date, very little is known for the intracellular events of QDs as siRNA carriers. Based on current findings, one can deduce that PEI molecule has high density of amine groups and they are able to exert proton sponge effect whereby promoting the inhibition of acidification of endosomal pH. This will leads to the net influx of chloride ions within the compartment, leading to the osmotic swelling and rupture of endosomal membrane, a phenomenon often called the “proton sponge effect” (Ramamoorth and Narvekar, 2015).

To be an efficient carrier, the applied nanoparticles must be biocompatible. The unique optical property of QDs makes them desirable for imaging and targeted delivery, but the presence of cadmium element in the QDs also creates great concern in the medical field since cadmium can cause strong toxicological impact to the body. Some *in vitro* studies have demonstrated that QDs are harmful to the cells if high doses of QDs are exposed and in general many of these QDs are not well passivated with coatings. Sung et al. revealed that long-term exposure to CdTe QDs to the cells can cause Cd^2+^ release and generating reactive oxygen species (ROS) which subsequently led to functional impairments to MCF-7 cell line (Cho et al., 2007). Similarly, Wang et al. reported that exposure to CdSe QDs resulted in cultured intestinal cell detachment and death (Wang et al., 2008). In practice, the cytotoxicity of QDs is based on several factors such as their inherent chemical composition, surface modification and charge (Hardman, 2006). If appropriate shell protection and surface coating are applied to the nanocrystals surface, the QDs can be made with low toxicity. Here, the CdSSe/ZnS QDs used are coated multilayer-shell, which increases the stability of QDs and decreases the cytotoxicity. After 48 h of treating the U87 and U251 cells with PEI modified CdSSe/ZnS QDs at 10 times of the transfected dose, no cytotoxicity sign was observed. These results indicated that QDs presented here is safe for transfection and can be applied for QDs-based gene delivery application.

In summary, we designed and prepared QD-PEI nanoformulation for efficient gene delivery and silencing, and the nanoplex developed here will serve as a promising candidate for gene therapy of central nervous system tumors. Although, the complete suppression of the cancer cell proliferation is still challenging, the development of the QD-PEI formulation in this study will certainly laid an important foundation in providing valuable information to guide future studies on gene therapy of cancers. With these inspiring results, we believe that further improvement in the QDs-based siRNA delivery strategy will allow one to achieve better application prospects in cancer gene therapy.

## Ethical approval

This article does not contain any studies with human participants or animals performed by any of the authors.

## Author contributions

GL, GX, and KY designed experiments. TC, JZ, YW, and YZ carried out experiments. JL, QH, and ZF assisted with sample collecting. XW and ML analyzed experimental results. GL wrote the manuscript and GX, KY revised the manuscript. All authors have contributed to the final version and approved the final manuscript.

### Conflict of interest statement

The authors declare that the research was conducted in the absence of any commercial or financial relationships that could be construed as a potential conflict of interest.
